# Structural Modeling of Cell Wall Peptidase CwpFM (EntFM) Reveals Distinct Intrinsically Disordered Extensions Specific to Pathogenic *Bacillus cereus* Strains

**DOI:** 10.3390/toxins12090593

**Published:** 2020-09-14

**Authors:** Seav-Ly Tran, Delphine Cormontagne, Jasmina Vidic, Gwenaëlle André-Leroux, Nalini Ramarao

**Affiliations:** 1 Micalis Institute, INRAE, AgroParisTech, Université Paris-Saclay, 78350 Jouy-en-Josas, France; seav-ly.tran@inrae.fr (S.-L.T.); delphine.cormontagne@inrae.fr (D.C.); jasmina.vidic@inrae.fr (J.V.); 2 MaIAGE, INRAE, AgroParisTech, Université Paris-Saclay, 78350 Jouy-en-Josas, France

**Keywords:** *Bacillus cereus*, cell wall peptidase, disordered extensions, homology modeling

## Abstract

The emergence of *B. cereus* as an opportunistic food-borne pathogen has intensified the need to distinguish strains of public health concern. The heterogeneity of the diseases associated with *B. cereus* infections emphasizes the versatility of these bacteria strains to colonize their host. Nevertheless, the molecular basis of these differences remains unclear. Several toxins are involved in virulence, particularly in gastrointestinal disorders, but there are currently no biological markers able to differentiate pathogenic from harmless strains. We have previously shown that CwpFM is a cell wall peptidase involved in *B. cereus* virulence. Here, we report a sequence/structure/function characterization of 39 CwpFM sequences, chosen from a collection of *B. cereus* with diverse virulence phenotypes, from harmless to highly pathogenic strains. CwpFM is homology-modeled in silico as an exported papain-like endopeptidase, with an N-terminal end composed of three successive bacterial Src Homology 3 domains (SH3b_1–3_) likely to control protein–protein interactions in signaling pathways, and a C-terminal end that contains a catalytic NLPC_P60 domain primed to form a competent active site. We confirmed in vitro that CwpFM is an endopeptidase with a moderate peptidoglycan hydrolase activity. Remarkably, CwpFMs from pathogenic strains harbor a specific stretch of twenty residues intrinsically disordered, inserted between the SH3b_3_ and the catalytic NLPC_P60 domain. This strongly suggests this linker as a marker of differentiation between *B. cereus* strains. We believe that our findings improve our understanding of the pathogenicity of *B. cereus* while advancing both clinical diagnosis and food safety.

## 1. Introduction

The cell wall (CW) of bacteria is an intricate mesh of macromolecules composed of peptidoglycan (PG), a complex polymer formed by linear glycan chains cross-linked by peptide stems. The glycan chains are usually long and alternate N-acetylglucosamine (NAG) with N-acetylmuramic acid (NAM) residues through β-(1, 4) bonds. In most Gram-positive bacteria like *Bacillus cereus*, the core structure of a non-cross-linked stem attached is L-Ala-*Ɣ*-D-Glu-L-Lys-D-Ala-D-Ala. Due to environmental adaptation, the PG core can undergo chemical modification like O-acetylation, N-deacetylation amidation or can incorporate Gly and non-canonical D-amino acids [1]. Additionally, the CW accommodates macromolecular components such as teichoic acid, lipoteichoic acids, polysaccharides and complex proteins like export secretion systems. CW integrity and plasticity need to be tuned to adapt readily to the bacterial cell cycle or to the changing environment, and its biosynthesis must be also highly regulated as CW disruption leads to bacterial cell death [2]. Accordingly, PG metabolism requires an exquisite and timely remodeling, organized by bacterial cell wall hydrolases (CWHs) [3]. CWHs play an essential physiological role in cell wall integrity. Additionally, they are implicated in bacterial adhesion and invasiveness [4], persistence in the host [5] and in the initiation step of biofilm formation [6], therefore contributing to bacterial pathogenicity. Moreover, the resulting cell wall fragments are recycled as signaling molecules to trigger bacterial communication, immune response or antibiotics resistance [5,7]. Finally, they also prime the insertion of supramolecular structures like secretion, flagella or pili systems [8]. Three types of CWH exist, each displaying a specificity towards PG: (i) cell wall amidase (CWA) catalyzes the hydrolysis of the amide bond between NAM and L-Ala at the N-terminal of the stem peptide; (ii) cell wall glycosidase (CWG) cleaves the glycosidic linkages; and (iii) cell wall peptidase (CWP) hydrolyses the amide bonds with the PG chains [8]. Recent structural data evidence that CWHs show modularity, with a catalytic domain combined to one or several CW binding domains (CBDs) located at the N- or C-terminal ends. This modular organization associated with CBD repeats is highly suspected to engage these hydrolases as a platform responsible for CW integrity. Interestingly, due to their inherent flexibility and lack of structural characterization, the linker regions that connect the binding domains to the catalytic one have received little attention until now. Nevertheless, interest is increasing as they are shown to play a role in domains orientation or swapping, and dynamics that result in substrate specificity and affinity [1,2,3].

The *Bacillus cereus* group is composed of rod-shaped, spore-forming, aerobic or facultative anaerobic species [9], among which the most noticeable members are species from *B. cereus sensu stricto* (usually referred to as *B. cereus*), *B. mycoides*, *B. pseudomycoides*, *B. weihenstephanensis*, *B. anthracis*, *B. thuringiensis*, *B. cytotoxicus* and *B. toyonensis*. The genetic proximity of the species within the *B. cereus* group has not permitted their distinction from a phylogenetic point of view; nevertheless, species can be differentiated through phenotypic characteristics, host species predilection and clinical manifestation [10,11]. Four members are pathogens, namely *B. thuringiensis, B. anthracis, B. cytotoxicus and B. cereus*. Despite being long considered as nonpathogenic to humans, *B. thuringiensis* has been occasionally identified as responsible for human infections including food poisoning-associated diarrheas, ocular infections, periodontitis, burn and wound infections or bacteremia [12]. In addition, *B. thuringiensis* can produce potent insecticidal crystal proteins and has been used since a decade ago as a bio-insecticide [13]. *B. anthracis* is the agent responsible for anthrax disease in animals and humans, and is an important biological warfare agent [14]. *B. cytotoxicus* is a human pathogen associated with occasional fatal food poisoning [15]. *B. cereus* is a major food-borne human pathogen (FBO), responsible for two types of food poisoning, the emetic and gastrointestinal syndromes [16]. The emetic syndrome is caused by an emetic toxin called cereulide, and results in vomiting [17,18,19,20]. The diarrheal syndrome is associated with three pore-forming enterotoxins, called Cytotoxin K (CytK1 and CytK2), Enterotoxin Nhe (non-hemolytic enterotoxin) and Enterotoxin Hbl (Hemolysin BL) [20,21]. Recently, the non-redundant Nhe and Hbl toxins were shown to share the same mode of action and to operate synergistically to activate the NLRP3 inflammasome, which is a critical component of host innate immune defense [22]. Other factors have also been implicated in *B. cereus* virulence. In particular hemolysins, proteases and repair factors have been shown to be involved in the resistance of *B. cereus* to the host immune system [23,24,25,26,27]. Furthermore, we have shown that EntFM, formerly isolated after purification from *B. cereus* strain FM-1 and identified as an enterotoxin [28], is in fact a cell wall peptidase. Accordingly, EntFM was renamed CwpFM [29]. CwpFM is a 45-kDa protein, responsible for characteristic enterotoxin symptoms in animal models, increases in vascular permeability in rabbits, infiltration into the ligated loops of rabbits and toxicity towards Vero epithelial cells, whereas it did not show either hemolytic or lecithinase activities [30,31]. Consistently, we have evidenced that CwpFM of *B. cereus* is involved in bacterial motility and morphology, adhesion to epithelial cells and biofilm formation and is essential to promote *B. cereus* virulence in an insect model of infection [27]. Prevalence studies have shown that the *cwpFM* gene is present on the chromosome as a single copy and restricted to almost all members of the *B. cereus* group [32,33,34].

Here, we report a sequence/structure/function characterization, following the subsequent bioinformatic analysis of 39 CwpFM sequences, chosen from a collection of *B. cereus* with a diverse virulence phenotype, from harmless to highly pathogenic strains. We also homology-modeled the 3D structure of the CwpFM protein from *B. cereus*. We show that CwpFM is an exported papain-like endopeptidase with, at the N-terminal end, three successive bacterial Src Homology 3 domains (SH3b_1-3_), and at the C-terminal end, a catalytic NLPC_P60 domain (New lipoprotein C/Protein of 60 KDa), that involves a competent active site. We confirmed in vitro that CwpFM is an endopeptidase despite a moderate peptidoglycan hydrolase activity. Additionally, we predicted that each of the three linkers connecting SH3b_1-2_, SH3b_2-3_ or SH3b_3_ to NLPC_P60 modules associates low-complexity with an intrinsically disordered pattern. Remarkably, CwpFMs from pathogenic strains harbor a specific stretch of intrinsically disordered linkers, particularly long, located between SH3b_3_ and catalytic NLPC_P60 domains. This pattern exquisitely discriminates pathogenic from non-pathogenic strains. Thus, we propose that it could define a marker of differentiation between the *B. cereus* strains.

## 2. Results

### 2.1. CwpFM is Present and Highly Conserved within the Members of the B. cereus Group

We have previously shown that CwpFM is only detected within the members of the *B. cereus* group [26]. Here, we did a comparative analysis of CwpFM from 10 strains representative of the *B. cereus* group (Table 1). A Blastn search with the complete published sequence of *cwpFM* from *B. cereus* isolate A6 (AY789084.1) was performed to identify CwpFM homologues in the selected genome (Table 2). 

Only one copy of the gene was detected in each *Bacillus* genome. All the proteins encoded by the identified CwpFM homologues belong to the C40 family peptidase. Our bioinformatic analysis revealed that CwpFM is highly conserved among the *B. cereus* group. Sequence identities, when compared to CwpFM of the A6 strain, range from 72% for *B. pseudomycoides* DSM 12442 to 99% for *B. cereus* ATCC 14579. The most closely related sequences are between *B. cereus* and *B. thuringiensis* species with identities above 95.5%.

### 2.2. Domain Organization of the CwpFM Homologues from the B. cereus Group Indicates the Presence of Cell Wall Degradation Domains

**Figure 1:**
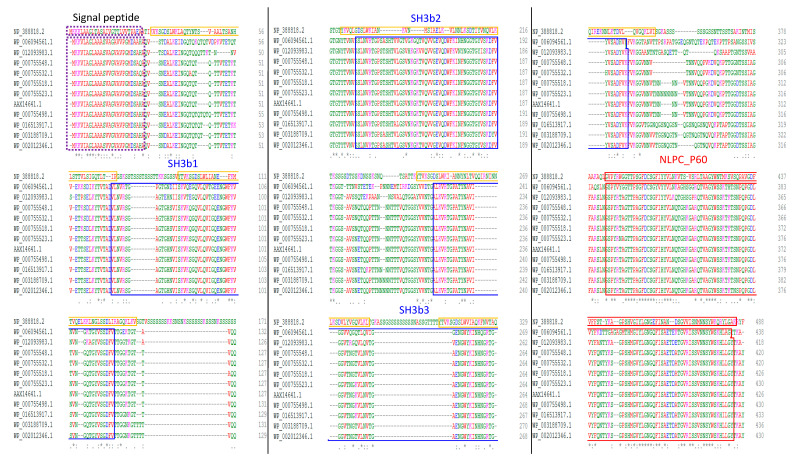
Multiple sequence alignment and domain organization of CwpFM homologues.

All the CwpFM amino acid sequences from strains representative of the *B. cereus* group were aligned and domains were identified using both Clustal Omega (clustalo 1.2.4) and InterPro online tools. A conserved signal peptide composed of 26 amino acid residues was predicted to translocate using the Sec translocon, and to be cleaved by Signal peptidase I at the cleavage site AH/QA_QV, located between positions 26 and 27. The prediction of secondary domains for CwpFMs indicated a modular topology combining three protein–protein interaction SH3b domains and one catalytic NLPC_P60 family for all the strains (Figure 1). This topology is predicted to be strictly conserved for all the strains, even for CwpFM from *B. pseudomycoides* that shows a sensitively lower identity with the other members of the *B. cereus* group. More largely, the topology is fully consistent with the modular architecture highlighted for CWPs in bacterial CWHs because it associates several cell wall binding domains to a catalytic domain [8].

Sequence alignment of CwpFM homologues from *B. cereus* isolate A6 (AAX14641.1), *B. cereus* ATCC 14579 (WP_000755498.1), *B. cereus ATCC* 10987 (WP_000755518.1), *B. thuringiensis* 407 cry- (WP_000755523.1), *B. thuringiensis* serovar konkukian str. 97–27 (WP_000755548.1), *B. mycoides* ATCC 6462 (WP_003188709.1), *B. pseudomycoides* DSM 12442 (WP_006094561.1), *B. weihenstephanensis KBAB4* (WP_002012346.1), *B. anthracis* str Ames (WP_000755532.1), *B. cytotoxicus* NVH 391–98 (WP_012093983.1) and *B. toyonensis* BCT-7112 (WP_016513917.1) show a similar domain organization (Figure 1). LytF of *B. subtilis* (NP_388818.2) was also aligned. Sequences were aligned using Clustal Omega (1.2.4) and domains were predicted using Interproscan (InterPro 78.1). The black box indicates the signal peptide, the blue boxes highlight SH3b domains and the red box shows the NLPC_P60 domain characteristic of the cell wall peptidases.

**Figure 2:**
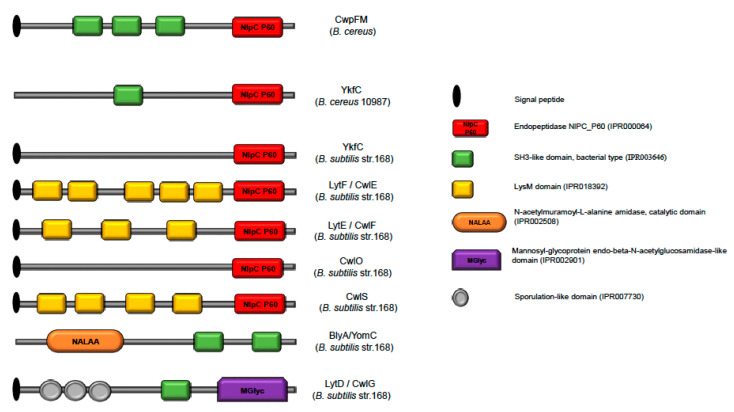
Schematic representation of cell wall hydrolases of *B. cereus* and *B. subtilis.* Domain organization of seven cell wall hydrolases from *B. subtilis* and two from *B. cereus*. InterProScan protein domain prediction analysis indicates the presence of a short signal peptide domain (black ellipse) at the N-terminus of every enzyme, except for YkfC (*B. cereus*) and BlyA (*B. subtilis*). Catalytic domains of three types can be distinguished: NLPC_P60 domain (red rectangle), N-acetylmuramoyl-L-alanine amidase, catalytic domain (orange rectangle) and Mannosyl-glycoprotein endo-beta-N-acetylglucosamidase-like domain (purple rectangle). The two cell-wall binding domains SH3b and LysM are shown in green and yellow squares, respectively. LytD has three sporulation-like domains specifically found in bacterial proteins involved in sporulation and cell division.

To gain further insights into the potential functions of CwpFM, the domain organization of CwpFM was compared with known NLPC_P60 proteins of *B. cereus* and *B. subtilis* (Figure 2). In all cases, a single NLPC_P60 domain is located at the C-terminus of the proteins. According to our domain prediction analysis, CwpFM and YkfC from *B. cereus* contain three and one SH3b domains, respectively, in addition to their catalytic domain NLPC_P60. However, the crystal structure of YkfC from *B. cereus* revealed that it actually contains two N-terminal SH3b domains [35]. By contrast, YkfC of *B. subtilis* has an NLPC_P60 domain, but neither any defined SH3b nor LysM Cell wall binding domain. Despite the strong biochemical characterization of YkfC in *B. cereus* and *B. subtilis*, there is no description of their physiological role in the bacteria. *B. subtilis* LytF shares 25% identity and 36% similarity with CwpFM of the ATCC 14579 *B. cereus* strain. When compared to CwpFM of the *B. cereus* group members, the LytF sequence differs in domain organization, evidencing a conserved NLPC_P60 domain at the C-terminus as in CwpFM, but no SH3b domain (Figure 1 and Figure 2). By contrast, LytF possesses five LysM domains. LytE, CwlO and CwlS of *B. subtilis* contain one NLPC_P60 domain and three, zero and four LysM domains, respectively. In any case, no SH3b domain was defined. This is consistent with the fact that the majority of cell wall peptidases display, in addition to their catalytic site, at least one domain necessary for the interaction with peptides, carbohydrates and lipids of the cell wall, such as SH3b or LysM domains. The endolysins BlyA (YomC) and LytD (CwlG) of *B. subtilis* also possess SH3b domains, but no NLPC_P60 domains. BlyA carries two SH3b domains linked to an N-acetylmuramoyl-L-alanine amidase catalytic domain. For the sake of clarity, the domain organization for known NLPC_P60 proteins of *B. cereus* and *B. subtilis* is resumed in Figure 2 below. Markedly, these differences in domain organization may reflect substrate specificities of the proteins, and functional and synergistic differences between *B. cereus* and *B. subtilis* CWHs.

### 2.3. Homology Modeling of CwpFM 3D Structure Highlights a Conserved Modular Topology Composed of Three SH3b Domains Connected to an NLPC_P60 Cysteine Peptidase Domain, and Emphasizes the Presence of Disordered Connecting Domains that Could Play a Role in Specificity and Affinity towards PG and Proteins

**Figure 3:**
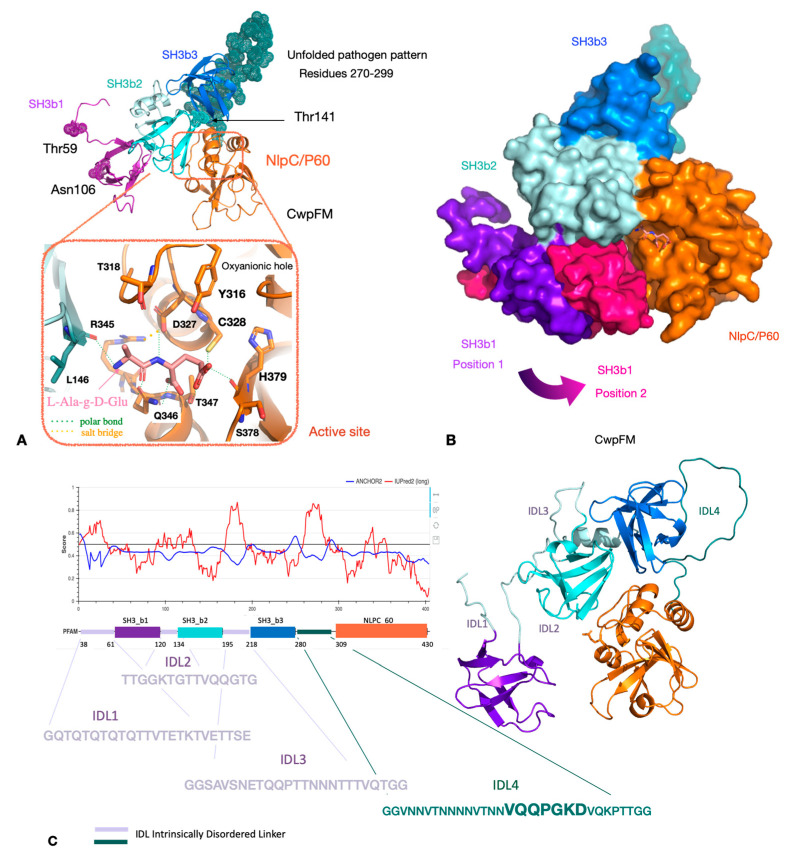
3D model of CwpFM from *B. cereus* ATCC 14579. Panel **A** upper view: the homology model of mature CwpFM highlights its modular topology with SH3b_1_ in purple (N-terminal end), SH3b_2_ in light blue, SH3b_3_ in marine blue and the catalytic NLPC_P60 domain in orange (C-terminal end). Panel A lower view: close view of the active site. The L-Ala-*Ɣ*-D-Glu ligand, accommodated in the active site, is shown as salmon sticks, and the residues of the active site are shown in light blue (SH3b_2_) and orange (NLPC_P60). Panel **B**: surface of CwpFM with the same color code as in A. The catalytic pocket is highlighted and the flexibility of SH3b_1_ is emphasized with two extreme positions, one in purple and the other in hot magenta, coming from two distinct homology models, and were superimposed on the catalytic domain. Panel **C**: details on the position, sequence and conformation of the insertions numbered IDL1, IDL2, IDL3 and IDL4. Figures were made by PyMOL, LLC, Schrödinger.

To highlight the main sequence/structure/function features of CwpFM within the *B. cereus* group, we focused on CwpFM full-length from *B. cereus* ATCC 14579 that contains 420 residues, the first 26 of which were confirmed to be a signal peptide by the Phobius server [35]. The mature sequence, i.e., the endopeptidase with the signal peptide excised, was predicted to be 37% similar to the putative dipeptidyl-peptidase VI from *Bacteroides ovatus* by HHpred (pdb id 3NPF). In addition, the N-terminal segment of CwpFM from *B. cereus* (amino acid residues 14–73) was predicted by HHpred to be 37% sequence-similar to the SH3b part of the putative endopeptidase from *Anabena variabilis* ATCC29413 (pdb id 2HBW) [36]. Since the similarity scores were high enough, we homology-modeled CwpFM from *B. cereus* with the above-cited templates. Expectedly, the homology model evidences a classical endopeptidase topology with three SH3b domains, named SH3b_1_, SH3b_2_ and SH3b_3_, located at the N-terminal-end, and the NLPC_P60 endopeptidase domain, located at the C-terminal end (Figure 3A,B). Both SH3b_2_ and NLPC_P60 contribute clearly to the formation of the active site, and possibly SH3b_1_, of which orientation varies most, up to 13 Å, according to the 100 models computed by the homology modeling. Interestingly, this could give a hint on the flexibility amplitude of this domain, with respect to the other domains (Figure 3B). However, modeling cannot infer on the possible swapping of SH3b domains, known to exist in CWPs, but hard to predict in silico. Thus, at this stage, one cannot thus exclude or certify any close interaction between SH3b_1,_ SH3b_2_ and NLPC_P60. The NLPC_P60 domain harbors a typical papain catalytic dyad, composed of strictly conserved Cys328 and His379 residues (Figure 3A). Additionally, it displays the strictly conserved Tyr316, known to act as the oxyanion hole, mandatory for endopeptidases. Further, sequence analysis evidences that both Tyr316 and Cys328 belong to the conserved catalytic motif YX_10_DCS. Thus, according to papain-like endopeptidases, the catalytic site of CwpFM is complete and prone to be active. Markedly, the sequence reveals large insertions from 17 to 34 residues, located at the N-terminus of each SH3b and between SH3b_3_ and NLPC_P60 domains (Figure 3C). Those insertions display low complexity with a large prevalence of polar Gln, Thr and Asn residues, and they are also highly susceptible to post-translational modifications and likely to be partially or completely unfolded. Accordingly, they have been named intrinsically disordered linkers and numbered from IDL_1_ to IDL_4_ (Figure 3C). Their role could be to adjust the positioning of each SH3b, either close to the active site for a functional role in specificity or remotely positioned to bind to the PG matrix or other cognate partners.

### 2.4. CwpFM Structure Is Able to Accommodate a PG Fragment

To ascertain, more precisely, the function of CwpFM, a L-Ala-*Ɣ*-D-Glu PG fragment, which corresponds to the reaction product performed by CWPs, was docked into the binding site formed at the interface between SH3b_2_ and NLPC_P60 domains (Figure 3B). The redocking of the L-Ala- *Ɣ*-D-Glu moiety inside YkfC shows a strictly similar accommodation, with respect to its crystal position, and a binding energy of −2.8 kcal/mol. The structure of *B. cereus* YkfC in complex with L-Ala-*Ɣ*-D-Glu was the first structural representative of an NLPC_P60 enzyme with a bound ligand. The enzyme releases L-Ala-*Ɣ*-D-Glu dipeptides from cell wall peptides via cleavage of an L-Ala- *Ɣ*-D-Glu-|-L-Lys bond.

The docking of L-Ala- *Ɣ* -D-Glu inside CwpFM gives an interaction energy of −3.7 kcal/mol, which is in the same range as in YkfC, thus suggesting that a short substrate with a free alanine residue could be accommodated in the active site of CwpFM. Additionally, the substrate accommodation highlights Tyr316, Asp327, Cys328, Ser329, Arg345, Gln346 and His379 (CwpFM numbering), as binding residues (Figure 3A). Interestingly, they are conserved in dipeptidyl-peptidase from *B. ovatus* and dipeptidyl-peptidase from *Anabaea variables* ATCC 29413. Particularly, Asp327 and Arg345 form a salt bridge strictly conserved in the three enzymes and involved in the hydrogen bonds network that connects many residues of the active site. Nevertheless, the binding shows a moderate affinity and the IDL extensions, which are difficult to model, could participate in reshaping the binding groove while enhancing the affinity. In short, CwpFM is able to accommodate the L-Ala- *Ɣ*-D-Glu product fragment of the peptidase reaction, but it is likely that a longer substrate with the L-Ala extremity, free or not, could also bind.

### 2.5. B. cereus CwpFM Shows a Weak PG Hydrolase Activity

**Figure 4:**
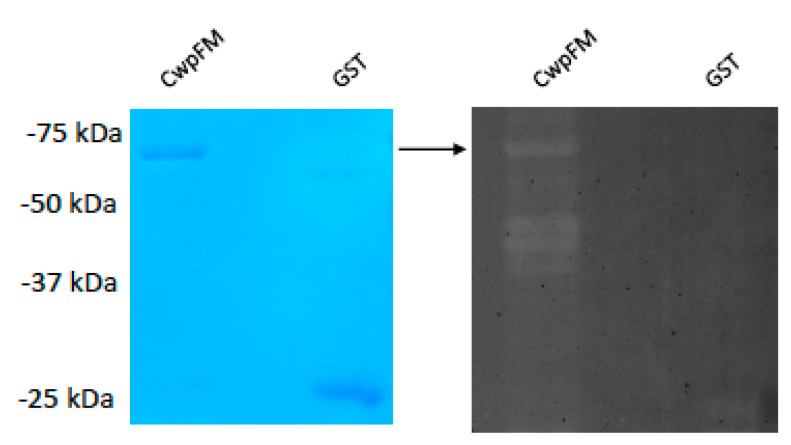
SDS-12%PAGE and zymography of CwpFM-GST. CwpFM-GST was overexpressed in the *E. coli* M15 strain. Lane M, protein standard; left panel: SDS-PAGE; right panel: zymography. The arrow indicates the position of the purified CwpFM-GST.

Our modeling of CwpFM identified a catalytic domain typical of cysteine papain peptidase and computed a reasonable affinity towards a PG moiety, so we aimed to characterize CwpFM PG hydrolase activity. We expressed and purified *B. cereus* CwpFM with a GST tag on the N-terminal end of the protein. Next, purified CwpFM enzyme activity was examined by zymography analysis using the *Micrococcus* cell wall as a substrate (Figure 4). The results showed a small clearance band in the zymogram. No clearing zones were observed when the same amount of purified GST was subjected to zymography. These results demonstrate that the CwpFM protein exhibits a weak CW degrading activity, which is consistent with our 3D model that illustrated a low binding affinity with PG.

### 2.6. CwpFM Distribution in B. cereus Strains of Various Pathogenic Potentials

We have shown that CwpFM is present and conserved amongst the *B. cereus* group members. However, within *B. cereus sensu stricto* strains, the pathologies vary from harmless to highly toxic strains. We thus studied CwpFM from *B. cereus* strains causing different pathologies: FBO, clinical non-gastrointestinal infections and non-pathogenic strains. We performed a homology search using the nucleotide sequence of *cwpFM* from *B. cereus* isolate A6 (AY789084.1) and we identified homologues of *cwpFM* as a single copy in every genome (Table 3), the result of which correlates with the high prevalence of the gene described in the literature. Despite being prevalent, these ORFs are variously annotated Peptidase P60, C40 family peptidase, putative endopeptidase LytE or Enterotoxin, in the databases. On average, the *cwpFM* genes showed between 89% and 99% identity. For the FBO and clinical strains, the *cwpFM* genes showed above 94% identity with the reference strain A6. The identity was, on average, lower for the non-pathogenic strains ranging from 89% to 92% identity, with two exceptions. These exceptions belong to the strains INRA PF (97% identity) and INRA A3 (99% identity). 

### 2.7. Analysis of the Differences at the 2D and 3D Levels

**Figure 5:**
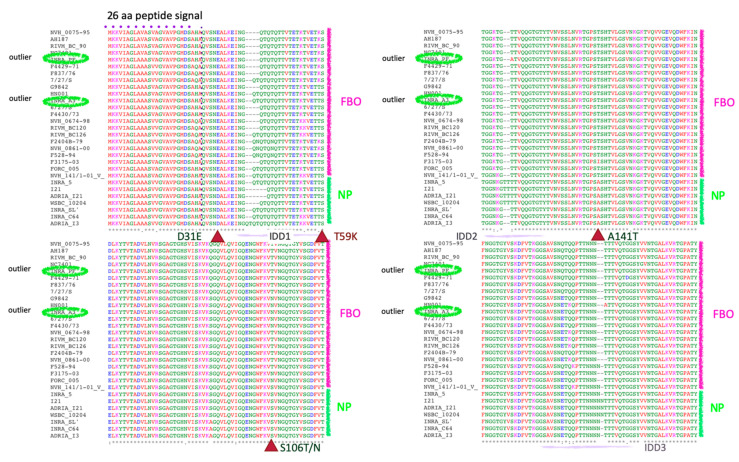

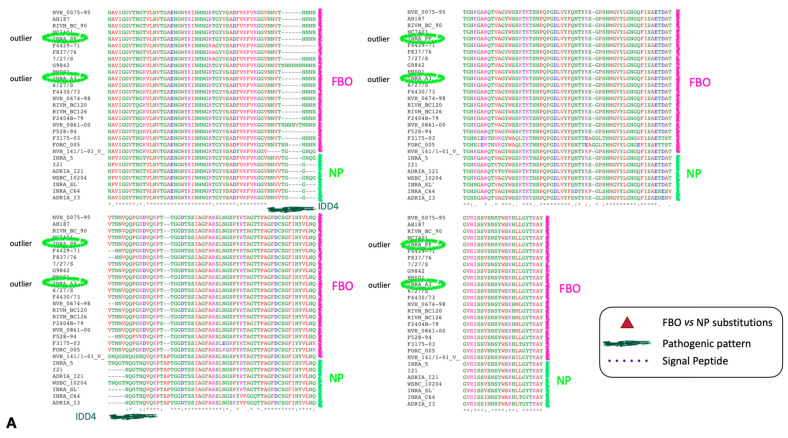

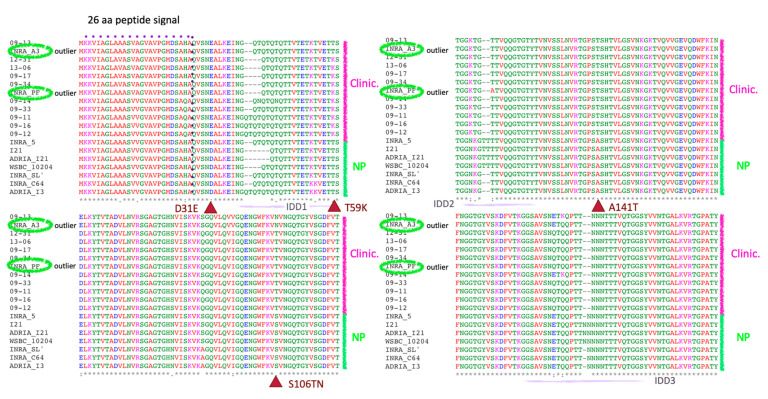

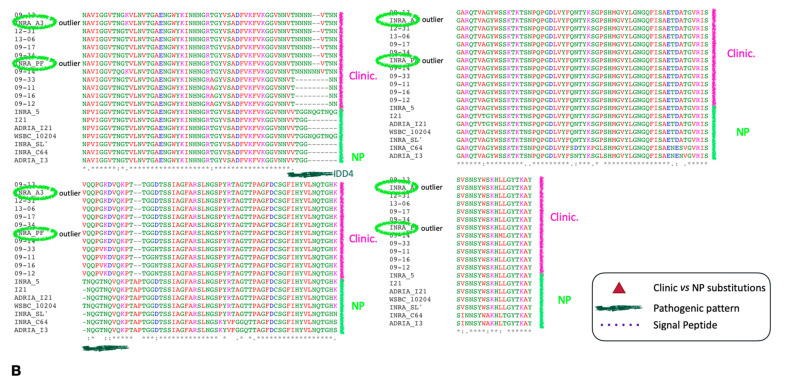
Alignment of CwpFM sequences of (**A**) NP vs. FBO strains and (**B**) NP vs. clinical strains. Panel **A**: CwpFM protein sequences of 9 non-pathogenic strains (NP) were aligned with those of 20 FBO strains and, in panel **B**: with 10 clinical strains. Alignments were performed using MAFFT. Amino acid substitutions strictly distinct between FBO or clinical and NP are indicated as red arrows.

Since we aimed to extract significant differences that could discriminate pathogenic from non-pathogenic strains, we align series of strain sequences using MAFFT. The first alignment is between FBO and non-pathogenic strains (Figure 5A), whilst the second is between clinical and non-pathogenic ones (Figure 5B).

The sequences of CwpFM cluster according to the origin of their *B. cereus* strain, with only two exceptions. Indeed, the CwpFM from the pathogenic strains, either FBO or clinical, are clearly separated from the non-pathogenic strains. The outsider strains INRA-PF and INRA-A3 clustered within the pathogenic strains group. Three point-mutations, Asp/Glu31, Thr/Asn106 and Thr/Ile141, and, more importantly, a 20-residues segment (276–296) located between SH3b_3_ and NLPC_P60 could be noted as significantly different between non-pathogenic strains and pathogenic strains. Glu31 is positioned at the N-terminal end, close to the excised peptide. Glu31 is substituted by an aspartic acid in non-pathogenic strains. Its substitution could not be mapped onto the CwpFM 3D structure because the protein was homology-modeled starting from residue 53. Thr/Asn106 in pathogenic strains is replaced by a serine, whilst Thr/Ile141 is substituted by an alanine, in non-pathogenic strains. In our homology model, Thr/Asn106 in SH3b_1_ and Thr/Ile151 in SH3b_2_ are positioned too far from each other to be in contact together (Figure 3A). Nevertheless, one can hypothesize that domain swapping or a close interaction between the two SH3b domains could favor the polar interaction between the two residues, the possibility of which cannot be excluded as domain swapping of SH3bs has been already mentioned for CWPs. Correspondingly, this feature could be claimed for the covariation of residues. Reversely, if we consider those residues as not engaged in mutual interaction, they could be largely accessible to any binding or post-translational modification. Markedly, the most significant difference is a segment, called IDL4, an intrinsically disordered linker, located between residues 280 and 309, that is clearly distinct in sequence when compared between non-pathogenic and pathogenic strains. Clearly, this segment could not be modeled (i) because it is partly an insertion as compared to our 3D templates, and (ii) because it is very low in complexity, and thus largely unstructured. Accordingly, in both non-pathogenic and pathogenic strains, we expect these segments of CwpFM to be natively unfolded. Notably, IDL4 displays a significant difference in sequences between non-pathogenic and pathogenic strains as VTGG(X)NQGTNQ (X being-, T or NQGTNQGT) is replaced by T(N)_0–6_VTNNVQQPGKD (Figure 3C and Figure 5).

The pathogenic strains harbor a significant amount of Asn residues, while the non-pathogenic ones display more Gly and Gln residues. Asn residues are highly susceptible to hydroxylation which is a post-translational modification shown recently as reversible [37]. Therefore, one cannot exclude the hydroxylation of Asn residues, which could contribute to protein modification, flexibility and anchoring at the PG. This is fully consistent with the linker role of IDL4 that connects SH3b_3_ to the catalytic NLPC_P60. Such modifications could tune the adequate positioning of SH3b_3_ towards protein partners, including SH3b_1_ and SH3b_2_, and adapt the exquisite mobility of the catalytic domain towards the PG. Due to the length and prevalence of Asn in all pathogenic strains, this segment could be considered as a pattern signature for *B. cereus* virulent strains.

## 3. Discussion

*B. cereus* is a serious cause of food poisoning. It is largely known that the emetic toxin and the enterotoxins Nhe, Hbl and CytK are responsible for vomiting and diarrhea syndromes, respectively [21,38]. Many other putative virulence factors have been described in *B. cereus*. However, their precise role in bacterial pathogenesis is still uncertain, although their involvement in virulence has been suggested due to their toxic effects on cellular models, insects or mammals. Unlike *B. anthracis*, *B. thuringiensis* and the specific *B. cereus* emetic strains, whose toxin genes are carried by plasmids, *B. cereus* virulence factors are specified by genes located on the chromosome and the virulence is probably multifactorial [20,38,39]. Among them, CwpFM, formerly identified as an enterotoxin, is in fact a cell wall peptidase of the NLPC_P60 family of peptidases, which is one of the most abundant secreted cell wall peptidase CWP families. Nevertheless, the CwpFM family lacks distribution, prevalence, sequence characterization and a molecular description of its mode of action.

Here, we report for the first time a distribution analysis of CwpFM within the *Bacillus* group. To infer the sequence/structure/function of CwpFM within the *B. cereus* group and gain molecular consistency, the features observed from bioinformatics analysis were mapped onto a 3D structure of CwpFM from *B. cereus* ATCC1479 modeled in silico. We highlight that CwpFM from *B. cereus* is a papain-like cysteine endopeptidase that displays the emblematic catalytic motif Y_316_X_10_DCS_329_ associated with the strictly conserved histidine residue H_379_ of the NLPC_P60 family. Cys_328_ of this motif and His_379_ residues form the catalytic dyad, while Y_316_ of the motif frames the oxyanionic hole, expected to occur in every peptidase protein. Thus, CwpFM displays a competent active site. Additionally, docking computation evidences that a PG moiety is able to bind to the active site. In line with that, structural bioinformatics analysis highlights that the binding site is composed of residues strictly conserved and located in both SH3b_2_ and NLPC_P60 domains. Only Gly_172_ in CwpFM replaces the bulky Tyr_118_ or Tyr_80_, and Gln_356_ replaces Asp_256_ or Asp_221_, in the YkfC of *B. cereus* and in the putative dipeptidyl-peptidase VI from *Bacteroides ovatus*, respectively. Since tyrosine and aspartate residues are known to interact together to fix the free amine group of the Alanine peptido-glycan moiety, the absence of a side chain in Gly_172_ could preclude the close interaction with Gln_346_ and result in an enlargement of the binding site while allowing the accommodation of substrates with an attached fragment at the Alanine extremity. Those structural features, key in the specific recognition of murein peptides by the subfamily of the NLPC_P60 protein, interrogate the substrate specificity and affinity. Accordingly, we could only detect in vitro a weak peptidoglycan hydrolase activity from purified CwpFM. One could argue that the PG from *Micrococcus lysodeikticus* ATCC M3770 may not be the cognate substrate, neither in PG length nor in molecular characteristics. One can also explain that the post-translational modification profile of CwpFM has not reached its optimum activity, due to its production in *E. coli,* that is unable to perform the hydroxylation of asparagine or phosphorylation of serine and threonine residues [40]. Still, the optimum substrate(s), the activation and the toxin target mode of action of CwpFM are open questions. We can also speculate that CwpFM activity is dependent of another enzyme/activator. As an example, it has been demonstrated that the amidase LytH of *Staphylococcus aureus* is only active in the presence of its membrane partner ActH [41]. With respect to the modular topology of CwpFM, the SH3b and catalytic domains could synergize to attain full endopeptidase activity. Further work is needed to identify the other substrates and/or activator allowing *B. cereus* CwpFM to be fully active, and to decipher the toxin mode of action.

*B. cereus* CwpFM contains three SH3b domains. Thus, despite its homology with *B. subtilis* LytF, the role of CwpFM probably differs from LytF. Indeed, the binding domains ensure the localization and the proper function of the CWH, particularly in CWPs [8]. Nine SH3b domains have been described (SH3b_-1_ to SH3b_-9_) so far and domains can be found at the two terminal ends of CWPs. Of note, cell wall binding domains that can be found along the mono-polypeptide chain generally improve the efficiency of the enzymatic activity, either by increasing the concentration of the ligand at the active site or by anchoring properly onto the enzyme, or even by reshaping the active site [1,8]. In line with that, the SH3b domains demonstrate a selective affinity for pentaglycine cross-bridges [42]. Additionally, it has been shown that loops from the SH3b domain can dock into the ends of the catalytic groove, remodel the substrate binding site and thus modulate substrate specificity [43]. This specificity can be driven by a single mutation. In line with that, two out of three mutations, that have been identified between non-pathogenic and pathogenic strains, are positioned on loops that could participate in substrate affinity or binding site reshaping. Further, it has recently been demonstrated that recognition is shared by two independent SH3bs, tightly engaged to each other, allowing protein clusterization [44,45]. Their interaction potentiates and compensates for the weak affinity of individual SH3b towards pentaglycine [42,46].

In *B. subtilis*, numerous CWPs such as LytF (CwlE), LytE (CwlF) and CwlS are known to work in synergy to ensure cell separation during vegetative growth, and deletion of these genes results in a long filament-like bacteria phenotype [47,48]. They all display an NLPC_P60 domain and a variable number of LysM (Lysin M) domains comprised between zero and five. LysM is amongst the most frequent CW binding motifs and has been shown to recognize the N-acetylglucosamine (GlcNac) polymers (NAG-X-NAG motif) of PG [49,50]. Particularly, LysM is involved in the specific localization of LytF at the separation sites and poles of *B. subtilis* [51]. LytE, in combination with CwlO, has also been associated with cell growth and morphogenesis as they both participate in breaking the PG cross-links along the lateral side of bacteria to support the process of elongation. Although domain prediction analysis reveals no presence of CW-binding domains within CwlO, it is most likely that a domain recognizing a specific fragment of the PG (probably LysM) is present to dictate the enzyme specificity of action. *B. subtilis* BlyA has three SH3b domains. To date, no physiological function has been linked to *B. subtilis* BlyA, however, BlyA from *Borrelia Burgdorferi* is a bacteriophage-encoded holin which, if expressed in *E. coli*, can induce damage to the *E. coli* cell envelope and allows the release of intracellular cytotoxin ClyA, inducing hemolysis on blood agar [52]. LytD (CwlG) was predicted to harbor one SH3b domain and a sporulation-like domain that may indicate a role during sporulation. Finally, LytD (CwlG) is an N-acetylglucosaminidase that has been implicated but is not essential in cell separation and motility [53]. Therefore, the exact role of LytD (CwlG) remains unclear.

CwpFM is present in all strains of our collection of *B. cereus*, gathering strains of various origins and causing different pathologies. CwpFM is a major *B. cereus* toxin that is involved in virulence. We have previously shown that CwpFM is involved in the bacterial shape and division, in adhesion to eukaryotic cells and in promoting virulence. Presence of the gene is now routinely assessed in combination with other diarrheal toxins-encoded genes such as *ces*, *nhe* and *hbl* to determine the potential pathogenicity of a strain. Data show that *cwpFM* is widely distributed (detection rate of 68–98%) in *B. cereus* isolated from diverse food matrices [53,54,55,56,57]. *cwpFM* is also detected in strains associated with food-borne illnesses [33,58] and is even present in emetic strains [59]. Due to the high distribution of *cwpFM* in pathogenic but also non-pathogenic strains, it is hard to use the detection of the *cwpFM* gene as a biomarker of pathogenicity. However, an accurate bioinformatics comparison between the sequences of our strain collection was performed, and then the residues distinct between non-pathogenic, FBO and clinical strains were mapped onto the homology model of CwpFM to check if the sequence and 3D structure could correlate with the pathogenicity of a strain [60]. All CwpFMs from *B. cereus* display four intrinsically disordered linkers (IDL) as connectors between SH3b and/or NLPC_P60 domains. Particularly, the IDL4 that connects SH3b_3_ to the catalytic domain is particularly long and displays a significant difference that both aggregates pathogenic FBO and clinical strains, while it segregates non-pathogenic strains. The stretch is NQGTNQVQ in non-pathogenic sequences that is replaced by the VQQPGKD patch in pathogenic ones. Additionally, one can observe an extra stretch of up to 10 additional asparagine residues found to be inserted in all pathogenic strains (FBO and clinical) and strictly absent in non-pathogenic ones. Such differences observed not only in length but also in the low complexity with a high prevalence of Asn (for pathogenic strains) vs Gly/Gln (for non-pathogenic strains) can have conformational and functional consequences. Interestingly, as this specific pattern defines the IDL4 junction domain between SH3b and NLPC_P60 domains, we suspect that this domain, natively unfolded, could play a role in the recruitment of binding partners, putatively through glycosylated Asn residues. The disordered part of CwpFM may possibly be involved in bacterial pathogenicity. Indeed, although deficient in stable secondary and tertiary structures under physiological conditions of pH and salinity, disordered parts in proteins may function as dynamic ensembles of interconverting conformers. Unstructured parts of CwpFM are depleted in hydrophobic amino acid residues, but enriched in polar and charged residues. It was shown that disordered proteins, enriched in polar/charged residues, are highly hydrated compared to ordered ones and as such, they behave distinctly in bulk and air/water or lipid/water interfaces [61]. For instance, α-synuclein, amyloid-β peptide and PB1-F2 influenza protein are disordered monomeric peptides in aqueous solution, but may adopt a β-sheet conformation that further aggregates into toxic amyloid fibrils in contact with negatively charged phospholipids and induces membrane morphological changes and disruption [62,63,64]. We previously reported the morphological changes of a mammalian cell membrane exposed to recombinant CwpFM [31], which may be linked to this region.

The precise role of CwpFM in pathogenic and non-pathogenic strains remains to be studied. The differences in sequences may have a direct or indirect role during virulence, possibly on the protein partners or post-translational modifications. This is a challenging question to address because many hydrolases, produced by the bacteria, may have a redundant function and take over in the case of a mutation. In addition, some have different functions and can even have more than one function [65]. For instance, in *Enterococcus faecalis*, the SagA protein is a secreted endopeptidase, which has an antibiotic role against enteric pathogens such as *Clostridium difficle* [66]. *Nocardia seriolae* protein NLPC_P60 is a cell wall peptidase also identified as a virulence factor [67]. In *Mycobacterium tuberculosis*, the protein Rv0024 has been shown to be involved in biofilm formation. Those biofilms have been found to be resistant to cell wall-acting anti-TB drugs [68]. Still, *M. tuberculosis*, a mutant lacking the NLPC_P60 protein, is more sensitive to antibiotics and lysozymes, leading to a decrease in the survival in macrophages [69], and the Rv2190c protein is required not only for cell wall maintenance but also for virulence since a mutant is less virulent in a mice model of infection in vivo [70]. Furthermore, NLPC_P60 was described as a virulence factor in *Bacillus anthracis* as it is part of its secretome and can be found in the blood of infected animals [71]. The function of CWP can also indirectly contribute to bacterial virulence. A recent work has demonstrated that the PG hydrolase Cwp19 contributes to *Clostridium difficile* autolysis, which induces the release of bacterial toxins [72]. Markedly, PG fragments released by CWPs can also act as signaling molecules to promote the presence of antimicrobial agents or to interact with the component of the host [73,74].

The versatility of these endopeptidases in the virulence of multiple bacteria towards the host could be explained by their modular architecture that also integrates intrinsically disordered segments and point mutations, both possibly subjected to post-translational modification. These data pave the way for further in silico and in vitro studies because they explore beyond the chromosomal gene presence and ground the first description of a pattern within *B. cereus* CwpFM sequences capable of discriminating pathogenic from non-pathogenic strains.

## 4. Materials and Methods

### 4.1. Bacterial Strain Sequences

The genome and *cwpFM* sequences of 10 strains belonging to the *B. cereus* group were retrieved from NCBI (Table 1). The genome and *cwpFM* sequences of 20 *B. cereus* strains isolated from food or human samples and associated with food poisoning were also retrieved from NCBI (Table 2). In addition, this study includes ten strains isolated from human samples following systemic or local infections [75] and nine non-pathogenic strains, isolated from food that did not cause infection in humans or animals [76,77]. The corresponding genome sequences were retrieved from the European Nucleotide Archive (ENA) or obtained in this study (Table 2).

### 4.2. Sequence Alignment, Conserved Motifs and Domain Analysis of the Proteins

Multiple nucleotide sequence alignments were conducted using the BLASTn interface. Multiple amino acid sequence alignments of the CwpFM proteins were performed using Clustal Omega v1.2.4 (https://www.ebi.ac.uk/Tools/msa/clustalo/). Signal peptide sequences were predicted using Phobius [36] and SignalP 5.0 [78] servers. The potential protein domains were identified using InterProScan protein domain prediction analysis (www.ebi.ac.uk/Tools/pfa/iprscan/).

### 4.3. D Alignments and Clustering of the Strains

Alignments were performed with MAFFT—Multiple Alignment using Fast Fourier Transform—v6.861b, with the default options [79]. It is a high-speed multiple alignment program which implements fast Fourier transform (FFT) to optimize protein alignments based on the physical properties of the amino acids. The program uses progressive and iterative alignment and is implemented at the ebi (https://www.ebi.ac.uk/Tools/msa/mafft/).

### 4.4. Molecular Modeling

CwpFM from the *B. cereus* ATCC 14579 strain (CwpFM_BC) was homology-modeled using the model-building software Modeller (mod9v18) [80]. The crystal structures of the apo putative dipeptidyl-peptidase VI from *Bacteroides ovatus* (pdb id 3NPF), and the SH3b domain of the putative endopeptidase from *Anabena variabilis* ATCC 29413 (pdb id 2HBW amino acid residues 14–73) served as 3D templates (10.2210/pdb3NPF/pdb; 10.2210/pdb2HBW/pdb) [35]. The 3D templates were previously chosen from the HHpred webserver, dedicated to structural homology detection. They were sorted as the two first hits. One hundred homology models of CwpFM were generated, and one was eventually chosen from a selection of the five best models with respect to the lowest values of the Modeller score function, best stereochemistry, as checked by Molprobity (http://molprobity.biochem.duke.edu/), and visual inspection, using PyMOL 2.0.7 (Schrödinger, LLC, New York, NY, USA). The selected model was then minimized using the Biologic suite of Schrödinger, LLC, New York, NY, USA. The sequences were also analyzed using IUPred to characterize disordered segments and identify molecular recognition features (https://iupred2a.elte.hu/) [81].

### 4.5. Docking

As a prerequisite before performing our docking of the L-Ala- *Ɣ*-D-Glu ligand in our model of CwpFM, we validated our protocol by redocking the ligand in the active site of YkfC (pdb id 3H41) because it is a structural and functional homologue in a holo configuration. Formerly, the ligand was discarded from YkfC to get the apo form and the tri-oxidized Cys238 of the crystal structure was reduced into a Cys residue to mimic the active site, with respect to the papain family of cysteine peptidases (pdb 3H41) [35]. Docking was performed using Autodock4 with the following parameters: a grid box centered on L-Ala- *Ɣ*-D-Glu bound to the catalytic site, encompassing all the residues involved in the interaction, a genetic algorithm of Lamarck, ten runs of computation with a final ranking and clustering of the docked peptide. The best pose computed for L-Ala- *Ɣ*-D-Glu superimposes very well to the L-Ala- *Ɣ*-D-Glu crystal position, so the protocol was validated and used for subsequent docking of L-Ala- *Ɣ*-D-Glu, in the apo form of a 3D template of dipeptidyl-peptidase VI from *Bacteroides ovatus* (pdb id 3NPF) and the homology-modeled CpwFM, after superimposition of their NLPC_P60 domains. The analysis of the complexes and figures were generated with PyMOL 2.0.7. (Schrödinger, LLC., New York, NY, USA).

### 4.6. Expression and Purification of the CwpFM-GST-Tagged Protein

The plasmid pGEX6P1-GST-CwpFM was constructed as follows. The *cwpFM* gene was amplified from the *B. thuringiensis 407* cry- chromosome by PCR using the primer pairs CwpFM-GST-1 (5′-CGGGATCCCAAGTTTCAAATGAAGCGCTAA-3′) and CwpFM-GST-2 (5′-CCGCTCGAGTCCCAAGTTTCCTTGGAAAGCC-3′). The DNA fragment was inserted between the BamHI and XhoI sites of plasmid pGEX6P1 (GE Healthcare), and the resulting plasmid was introduced into *E. coli M15* [pREP4] (Qiagen). Additionally, *E. coli BL21* bacteria were transformed with pGEX6P1–GST plasmid. Strains were grown at 37 °C for 8 h in 100 mL of LB containing ampicillin (100 μg/mL) for the strain BL21 or ampicillin and kanamycin (40 µg/mL) for the strain M15. Protein expression was induced by adding 100 μg/mL isopropyl-β-d-thio-galactoside for 4h at 30 °C. For the purification of the recombinant GST and CwpFM-GST fusion, bacterial pellets were re-suspended in lysis buffer (50 mm Tris-HCl, pH 7.8, 60 mm NaCl, 1 mm EDTA, 2 mm DTT, 0.2% Triton X-100) supplemented with complete Protease Inhibitor mixture (Roche), and incubated for 1 h on ice. Cells were then disrupted using a BAZIC Z cell disruptor (Constant Systems Ltd., Daventry, UK) at a pressure of 1600 bars, and centrifuged at 4 °C for 30 min at 10,000× *g*. GSH-Sepharose 4B beads (GE Healthcare) were added to clarified supernatants and incubated at 4 °C for 3 h. Beads were then washed two times in lysis buffer and three times in 20 mM Tris buffer, pH 8. Purity was assessed by 12% (v/v) SDS-PAGE with Coomassie blue staining. Protein concentration was measured using a Bradford assay (Sigma France, Lezennes, France).

### 4.7. Zymogram

CwpFM cell wall hydrolyzing activity was assessed using zymogram analysis. An amount of 3.5 µg of purified GST and CwpFM-GST was loaded onto SDS-PAGE using 12% (v/v) polyacrylamide separating gels containing 0·2% (v/v) *Micrococcus lysodeikticus* ATCC M3770 (Sigma) as the enzyme substrate. Micrococci were suspended in water and heat-inactivated at 120 °C for 10 min before they were mixed into the resolving gel. After sample migration, gels were washed with deionized water for 1 h at room temperature and incubated in 50 mM Tris-HCl pH 8 containing 1% Triton X100 (v/v) for 24 h at 37 °C. The total amount of proteins was detected by staining of the SDS-PAGE gel with Coomassie blue staining. The CwpFM hydrolyzing activity was characterized by a lysis plague visible as a halo on the gel at the level of the protein.

## Figures and Tables

**Table 1:** Distribution of CwpFM within the *Bacillus cereus* group. Characteristics of representative strains of the *Bacillus cereus* group.

Species	Strain	Origin/Description	PanC Group	Ref Genome
*B. cereus*	ATCC 14579	Isolated from a farmhouse in the United States, 1916	IV	NC_004722.1
*B. cereus*	ATCC 10987	Isolated from dairy cheese in Canada, 1930	III	NC_003909.8
*B. thuringiensis*	Bt407 cry-	Soil isolate that has been cured of the plasmid that encodes the intesticidal crystalline toxin	IV	NC_018877.1
*B. thuringiensis*	serovar konkukian str. 97–27	Soil organism isolated from a severe tissue necrosis of a soldier severely wounded by a land mine explosion in former Yugoslavia, 1995	III	NC_005957.1
*B. mycoides*	ATCC 6462	Also known as DSM 2048; isolated from soil	VI	NZ_CP009692.1
*B. pseudomycoides*	DSM 12442	Also known as NRRL B617; isolated from soil in Ghana, 1998	I	NZ_CM000745.1
*B. weihenstephanensis*	KBAB4	Psychrotolerant soil isolate isolated in forest soil in France, 2000	VI	NC_010184.1
*B. anthracis*	Ames	Isolated from a dead cow in Texas, 1981	III	NC_007530.2
*B. cytotoxicus*	NVH 391-98	Isolated from a food poisoning outbreak (vegetable puree) in a nursing home for elderly people in France, 1998	VII	NC_009674.1
*B. toyonensis*	BCT-7112	Isolated for use as probiotics in animal nutrition in Japan, 1966	V	NC_022781.1

**Table 2:** Blastn of *B. cereus* isolate A6 *cwpFM* nucleotide sequence (AY789084.1) against the genome of representative strains of the *Bacillus cereus* group.

Species	Strain	Identities	Gaps	Strand	Gene Identification	Protein Name	Protein Reference	Max Score	Total Scorer	Query Cover	E Value	Per. Ident.
*B. cereus*	ATCC 14579	1277/1293 (99%)	12/1293 (0%)	Plus/Plus	BC_RS09755	C40 family peptidase	WP_000755498.1	838	838	1.00	0.0	99.07%
*B. cereus*	ATCC 10987	1238/1281 (97%)	0/1281 (0%)	Plus/Plus	BCE_RS10070	C40 family peptidase	WP_000755518.1	828	828	1.00	0.0	97.89%
*B. thuringiensis*	Bt407 cry-	1268/1293 (98%)	12/1293 (0%)	Plus/Plus	BTB_RS09950	C40 family peptidase	WP_000755523.1	836	836	1.00	0.0	98.84%
*B. thuringiensis*	serovar konkukian str. 97-27	1215/1281 (95%)	18/1281 (1%)	Plus/Plus	BT9727_RS09585	C40 family peptidase	WP_000755548.1	800	800	1.00	0.0	95.54%
*B. mycoides*	ATCC 6462	1178/1312 (90%)	32/1312 (2%)	Plus/Minus	BG05_RS21755	C40 family peptidase	WP_003188709.1	667	667	1.00	0.0	88.99%
*B. pseudomycoides*	DSM 12442	997/1324 (75%)	47/1324 (3%)	Plus/Plus	BPMYX0001_RS08705	C40 family peptidase	WP_006094561.1	582	582	0.99	0.0	72.27%
*B. weihenstephanensis*	KBAB4	1168/1296 (90%)	18/1296 (1%)	Plus/Plus	BCERKBAB4_RS09410	C40 family peptidase	WP_002012346.1	672	672	1.00	0.0	91.44%
*B. anthracis*	Ames	1211/1281 (95%)	18/1281 (1%)	Plus/Plus	GBAA_RS09755	C40 family peptidase	WP_000755532.1	796	796	1.00	0.0	95.07%
*B. cytotoxicus*	NVH 391-98	1050/1290 (81%)	39/1290 (3%)	Plus/Plus	BCER98_RS07835	C40 family peptidase	WP_012093983.1	644	644	0.99	0.0	81.65%
*B. toyonensis*	BCT-7112	1200/1309 (92%)	35/1309 (2%)	Plus/Plus	BTOYO_RS22750	C40 family peptidase	WP_016513917.1	711	711	1.00	0.0	91.45%

**Table 3:** Characterization of *B. cereus* strains used in this study and their corresponding CwpFM proteins.

Strain	Collection	Origin/Description	Ref Genome	Identities	Gaps	Strand	Gene Identification	Protein Name	Protein Reference
NVH 0075/95	FBO	Stew with vegetables, food poisoning outbreak of diarrheal syndrome in Norway, 1995	LABM00000000.1	1236/1281(96%)	0/1281(0%)	Plus/Plus	TU63_19225	peptidase P60	KMP84856.1
G9842	FBO	Human stool, outbreak that involved three individuals in the USA, 1996	NC_011772.1	1273/1299(98%)	18/1299(1%)	Plus/Plus	BCG9842_RS09225	C40 family peptidase	WP_000755522.1
AH187	FBO	Human vomit of a person having previously eaten cooked rice in London, emetic outbreak in UK, 1972	NC_011658.1	1236/1281(96%)	0/1281(0%)	Plus/Plus	BCAH187_RS10030	C40 family peptidase	WP_000755553.1
NC7401	FBO	Feces/vomit, food poisoning in Japan, 1994	NC_016771.1	1234/1281(96%)	0/1281(0%)	Plus/Plus	BCN_RS09705	C40 family peptidase	WP_014297774.1
NVH_141/1-01_V_C53	FBO	Vegetarian pasta, diarrheal food poisoning outbreak in Norway, 2001	FMJK00000000.1	1191/1299(92%)	27/1299(2%)	Plus/Plus	BC141101_01248	Enterotoxin	SCN16078.1
NVH 0674-98	FBO	Mashed swedes/scrambled eggs, diarrheal food poisoning in Norway, 1998	FMJM00000000.1	1231/1281(96%)	6/1281(0%)	Plus/Plus	BC067498_01849	Enterotoxin	SCN44411.1
HN001	FBO	Human vomit, food poisoning in China, 2000	NZ_CP011155.1	1272/1287(99%)	6/1287(0%)	Plus/Plus	WR52_RS09190	C40 family peptidase	WP_063536128.1
NVH 0861-00	FBO	Ice scream, diarrheal food poisoning in Norway, 2000	FMBJ00000000.1	1221/1305(94%)	27/1305(2%)	Plus/Plus	BC0861_01953	Enterotoxin	SCC09072.1
RIVM_BC120	FBO	Human feces, diarrheal food poisoning in Netherlands	FMIJ00000000.1	1237/1281(97%)	0/1281(0%)	Plus/Plus	BCRIVMBC120_02096	Enterotoxin FM	SCL92408.1
RIVM_BC126	FBO	Human feces, diarrheal food poisoning in Netherlands	FMJJ00000000.1	1238/1287(96%)	6/1287(0%)	Plus/Plus	BCRIVMBC126_01928	Enterotoxin	SCN07222.1
F2404B-79	FBO	Diarrheal food poisoning outbreak in the UK	FMJG00000000.1	1231/1287(96%)	6/1287(0%)	Plus/Plus	BCF24048_01956	Enterotoxin	SCM94734.1
6/27/S	FBO	Human feces, diarrheal	NZ_LABV00000000.1	1278/1281(99%)	0/1281(0%)	Plus/Plus	TU48_RS34500	C40 family peptidase	WP_000755525.1
F3175/03(D7)	FBO	Human feces, diarrheal	NZ_JYPI00000000.1	1214/1289(94%)	13/1289(1%)	Plus/Plus	TU54_28630	peptidase P60	KMP31545.1
F528/94	FBO	Poisoning outbreak from beef and chow rice in the UK, 1994	NZ_JYPH00000000.1	1207/1290(94%)	27/1290(2%)	Plus/Plus	TU52_12565	peptidase P60	KMP35524.1
F4429/71	FBO	Vanilla pudding, diarrheal	NZ_JYPJ00000000.1	1214/1281(95%)	18/1281(1%)	Plus/Minus	TU55_10935	peptidase P60	KMP45116.1
RIVM BC 90	FBO	Human feces, diarrheal, 1999	LABN00000000.1	1236/1281(96%)	0/1281(0%)	Plus/Plus	TU64_27355	peptidase P60	KMP79201.1
7/27/S	FBO	Human feces, diarrheal	NZ_LABW00000000.1	1232/1287(96%)	6/1287(0%)	Plus/Plus	TU49_15830	peptidase P60	KMP18977.1
FORC_005	FBO	Korean side dish, food-borne illness in South Korea	NZ_CP009686.1	1215/1301(93%)	25/1301(1%)	Plus/Plus	FORC5_RS09925	C40 family peptidase	WP_044307235.1
F4430/73	FBO	Peas soup, diarrheal syndrome in Belgium, 1973	JYPK00000000.1	1278/1281(99%)	0/1281(0%)	Plus/Minus	TU56_09675	peptidase P60	KMP71144.1
F837/76	FBO	Food-borne outbreak in the UK, 1976	NC_016779.1	1214/1281(95%)	18/1281(1%)	Plus/Plus	BCF_RS09455	C40 family peptidase	WP_000755546.1
09–13	Clinical	Premature newborn, blood culture, 2009	this study	1277/1287 (99%)	6/1287 (0%)	Plus/Plus		putative peptidoglycan endopeptidase LytE	
09–14	Clinical	Premature newborn, blood culture, 2009	this study	1235/1293 (96%)	12/1293 (0%)	Plus/Plus		putative peptidoglycan endopeptidase LytE	
09–33	Clinical	New born, axilla, 2009	ERS1507218	1215/1287 (94%)	24/1287 (1%)	Plus/Plus		putative peptidoglycan endopeptidase LytE	
12–31	Clinical	Premature newborn, blood culture, 2011	this study	1236/1281 (96%)	0/1281 (0%)	Plus/Plus		putative peptidoglycan endopeptidase LytE	
13–06	Clinical	Intensive care unit, blood culture from catheter, 2011	this study	1236/1281 (96%)	0/1281 (0%)	Plus/Plus		putative peptidoglycan endopeptidase LytE	
09–11	Clinical	Premature newborn, blood culture, 2009	ERS1493302	1215/1293 (94%)	30/1293 (2%)	Plus/Plus		putative peptidoglycan endopeptidase LytE	
09–16	Clinical	New born, Umbilicus, 2009	ERS1494027	1215/1293 (94%)	30/1293 (2%)	Plus/Plus		putative peptidoglycan endopeptidase LytE	
09–12	Clinical	Premature newborn, cerebrospinal fluid, 2009	ERS1494026	1215/1293 (94%)	30/1293 (2%)	Plus/Plus		putative peptidoglycan endopeptidase LytE	
09–17	Clinical	Surface of neonatology ward (window sill), 2009	ERS1494028	1236/1281 (96%)	0/1281 (0%)	Plus/Plus		putative peptidoglycan endopeptidase LytE	
09–34	Clinical	Premature newborn, stomach tube feeding, 2009	ERS1494031	1236/1281 (96%)	0/1281 (0%)	Plus/Plus		putative peptidoglycan endopeptidase LytE	
INRA PF	Non-pathogenic	Milk protein	this study	1237/1281 (97%)	0/1281 (0%)	Plus/Plus		putative peptidoglycan endopeptidase LytE	
INRA 5	Non-pathogenic	Pasteurized zucchini puree	FLZU01000000	1187/1309 (91%)	29/1309 (2%)	Plus/Plus		putative peptidoglycan endopeptidase LytE	
INRA C64	Non-pathogenic	Pasteurized vegetables	this study	1157/1294 (89%)	29/1294 (2%)	Plus/Plus		putative peptidoglycan endopeptidase LytE	
ADRIA I3	Non-pathogenic	Cooked foods	this study	1156/1300 (89%)	35/1300 (2%)	Plus/Plus		putative peptidoglycan endopeptidase LytE	
INRA A3	Non-pathogenic	Starch	LABH01000000	1277/1287 (99%)	6/1287 (0%)	Plus/Plus		putative peptidoglycan endopeptidase LytE	
INRA SL’	Non-pathogenic	Soil	this study	1184/1290 (92%)	21/1290 (1%)	Plus/Plus		putative peptidoglycan endopeptidase LytE	
I21	Non-pathogenic	Unknown	this study	1170/1293 (90%)	30/1293 (2%)	Plus/Plus		putative peptidoglycan endopeptidase LytE	
ADRIA I21	Non-pathogenic	Cooked foods	FMJF01000000	1170/1293 (90%)	30/1293 (2%)	Plus/Plus		putative peptidoglycan endopeptidase LytE	
WSBC 10204	Non-pathogenic	Pasteurized milk	PRJNA258373	1187/1306 (91%)	26/1306 (1%)	Plus/Plus		putative peptidoglycan endopeptidase LytE	
